# Endonuclease IV Is the Major Apurinic/Apyrimidinic Endonuclease in *Mycobacterium tuberculosis* and Is Important for Protection against Oxidative Damage

**DOI:** 10.1371/journal.pone.0071535

**Published:** 2013-08-01

**Authors:** Rupangi Verma Puri, Nisha Singh, Rakesh K. Gupta, Anil K. Tyagi

**Affiliations:** Department of Biochemistry, University of Delhi South Campus, New Delhi, India; Infectious Disease Research Institute, United States of America

## Abstract

During the establishment of an infection, bacterial pathogens encounter oxidative stress resulting in the production of DNA lesions. Majority of these lesions are repaired by base excision repair (BER) pathway. Amongst these, abasic sites are the most frequent lesions in DNA. Class II apurinic/apyrimidinic (AP) endonucleases play a major role in BER of damaged DNA comprising of abasic sites. *Mycobacterium tuberculosis*, a deadly pathogen, resides in the human macrophages and is continually subjected to oxidative assaults. We have characterized for the first time two AP endonucleases namely Endonuclease IV (End) and Exonuclease III (XthA) that perform distinct functions in *M.tuberculosis*. We demonstrate that *M.tuberculosis* End is a typical AP endonuclease while XthA is predominantly a 3′→5′ exonuclease. The AP endonuclease activity of End and XthA was stimulated by Mg^2+^ and Ca^2+^ and displayed a preferential recognition for abasic site paired opposite to a cytosine residue in DNA. Moreover, End exhibited metal ion independent 3′→5′ exonuclease activity while in the case of XthA this activity was metal ion dependent. We demonstrate that End is not only a more efficient AP endonuclease than XthA but it also represents the major AP endonuclease activity in *M.tuberculosis* and plays a crucial role in defense against oxidative stress.

## Introduction

Cellular DNA is subjected to continuous assaults by a wide variety of intimidating endogenous and exogenous agents. Amongst these, the predominant damage is caused by reactive oxygen species (ROS) that are formed as by-products of oxidative metabolism in organisms with aerobic respiration. Exogenous agents such as chemical carcinogens and ionizing radiation also generate ROS. These oxygen radicals mostly produce non-bulky DNA lesions that are substrates for the base excision repair (BER) pathway [1], [2]. This pathway is highly conserved from bacteria to humans [3]. Apurinic and apyrimidinic (AP) sites (also known as abasic sites), are the most common DNA lesions that arise during the spontaneous loss of normal or damaged bases or by the action of DNA glycosylases that release modified or mismatched bases from DNA [4], [5], [6]. The AP sites threaten genetic stability because they block replication and are mutagenic [7], [8]. Thus, the removal of AP sites in DNA by a Class II AP endonuclease is a crucial step in BER. AP endonuclease enzymes initiate the repair of abasic sites in DNA by cleavage of the DNA backbone immediately 5′ of an AP residue. A single-nucleotide gap is then created in the DNA by the action of 5′ deoxyribose phosphodiesterase. The base excision repair is completed by resynthesis of the DNA by DNA polymerase followed by the action of DNA ligase [9].

The Class II AP endonucleases have been classified into two families, the exonuclease III (ExoIII) and endonuclease IV (EndoIV) families, based on their homology to the two *Escherichia coli* enzymes. In *E.coli* the major AP endonuclease is ExoIII (Ec-ExoIII) representing 90% of the cellular AP endonuclease activity, while EndoIV (Ec-EndoIV) accounts for 10% of the total activity [10]. The *E.coli* AP endonucleases also exhibit additional 3′ phosphatase and 3′ phosphodiesterase activities in common. These activities are responsible for removing a multitude of blocking groups, including 3′ phosphate and 3′ phosphoglycolate, that are present at single-stranded breaks in DNA, induced by oxidative agents [11], [12]. In addition, Ec-EndoIV and Ec-ExoIII exhibit 3′→5′ exonuclease activity. *Saccharomyces cerevisiae* also possesses two AP endonucleases, the Apn1 and Apn2 proteins that represent the EndoIV and the ExoIII family, respectively [13]. However, the major AP endonuclease in this organism is Apn1, that exhibits a strong AP endonuclease activity in yeast cells while the Apn2 protein, is a weak AP endonuclease that exhibits strong 3′→5′ exonuclease and 3′ phosphodiesterase activities [13], [14], [15], [16]. Contrary to the above discovery in budding yeast, in the fission yeast *Schizosaccharomyces pombe*, Apn2 provides the major AP endonuclease activity while the Apn1 serves only as a back up activity [17]. The human AP endonucleases, Ape1 and Ape2, are both members of the ExoIII family where Ape1 is the major human AP endonuclease, while EndoIV homologs are not known in humans [18]. Amidst this vast heterogeneity observed amongst different species with respect to the function(s) of AP endonucleases, in the current study, we were interested in deciphering the role of AP endonucleases in mycobacteria.

*Mycobacterium tuberculosis*, is a highly successful pathogen, killing approximately 1.4 million infected people annually [19]. In the host, this bacterium resides in macrophage in an environment rich in reactive oxygen and nitrogen intermediates capable of damaging its genome [20], [21]. To maintain its genome integrity, the bacterium must possess robust DNA repair machinery. Further, the GC rich (∼66%) genome of this pathogen renders it much more susceptible to cytosine deamination (generating uracil) and guanine oxidation [predominantly generating 8-oxoguanine (8-oxoG)] than other intracellular bacteria. This has led to special interest in the BER pathways that repair uracil [22] and 8-oxoG [23] in mycobacteria and it is thought that BER may play a central role in maintaining the integrity of DNA in this bacterium in the absence of any recognized homologs of mismatch repair [6], [24]. However, in spite of the predicted importance of base excision repair to this pathogen [6], [25], studies in BER have been primarily restricted to biochemical and functional characterization of DNA glysosylases such as uracil DNA glycosylase (Ung,UdgB) [22], [26], formamidopyrimidine DNA glycosylase (MutM) [27], [28], [29] and adenine DNA glycosylase (MutY) [23]. The AP sites that result from the action of DNA glycosylases are cytotoxic and mutagenic. In fact, the inability to repair these AP sites is detrimental to the survival in the host as seen in various pathogens such as *Salmonella*, *Neisseria*, *Toxoplasma* etc. [30], [31], [32]. However, to the best of our knowledge no studies have focused on the role and characterization of AP endonucleases in *M.tuberculosis*. An understanding about the role of these enzymes in *M.tuberculosis* would shed light on the repair processes that this pathogen employs to counter the host defense mechanism(s) and help evolve strategies to combat TB.

The sequencing of *M.tuberculosis* genome revealed the presence of Ec-EndoIV and Ec-ExoIII homologs namely Endonuclease IV (End) and Exonuclease III (XthA), that are encoded by the genes *end* (Rv0670) and *xthA* (Rv0427c), respectively [33]. In the current study, for the first time, biochemical and functional characterization of these proteins has been carried out in *M.tuberculosis*.

## Materials and Methods

### Bacterial Strains and Growth Conditions

*E.coli* XL-1Blue (Stratagene) and BL21 (λDE3) (Novagen) strains were employed for cloning and protein expression, respectively. *E.coli* was propagated in Luria-Bertani (LB) media. Mycobacterial strains were grown on Middlebrook 7H11 (MB7H11) agar supplemented with 10% oleic-acid albumin dextrose catalase (OADC) or in MB7H9 medium supplemented with 10% ADC, 0.2% glycerol, and 0.05% Tween 80 at 37°C with shaking at 200 rpm. Recombineering method [34] was employed to generate mutants of *end* (MtbΔ*end*), *xthA* (MtbΔ*xthA*) and both the genes together (MtbΔ*end*Δ*xthA*) in *M.tuberculosis* H37Rv. *end* and *xthA* mutants were complemented extra-chromosomally on a plasmid to generate complements MtbΔ*end*Comp and MtbΔ*xthA*Comp, respectively. The strains were confirmed by immunoblotting (Puri, R.V. *et. al*, unpublished data). Ampicillin was used at a concentration of 50 µg/ml for *E.coli*. Kanamycin and chloramphenicol were used at concentrations of 25 µg/ml and 30 µg/ml, respectively. Hygromycin was used at a concentration of 50 µg/ml for mycobacteria or at 150 µg/ml for *E.coli*.

### Expression of *end* and *xthA* and Purification of the Encoded Proteins

The AP endonuclease genes *end* (Rv0670) and *xthA* (Rv0427c) were PCR amplified by using *M.tuberculosis* H37Rv genomic DNA as the template by using primers (Table S1) designed from the published genome sequence. The primers were designed such that the expressed proteins comprise of a streptavidin tag at their C-terminus. PCR amplified *end* and *xthA* were separately cloned into the pet-21c(+) expression vector (Novagen) by using *Nhe*I and *Hind*III restriction sites to generate pet-21c-*end* and pet-21c-*xthA* constructs, respectively. The constructs were confirmed by DNA sequencing.

*E.coli* BL21 (λDE3) cells were separately transformed with pet-21c-*end* and pet-21c-*xthA* **.** Cells were grown to an A_600 nm_ of 0.8 in LB media containing 50 µg/ml of ampicillin and synthesis of proteins was induced by the addition of 1 mM isopropyl-1-thio-β-D-galactopyranoside (IPTG) followed by incubation at 18°C for 18 hours with constant shaking at 200 rpm. Cells were resuspended in 20 mM Tris-HCl (pH 8.0), containing 50 mM NaCl, 1 mM phenylmethylsulfonyl fluoride (PMSF) and 2 mM β-mercaptoethanol (buffer A) followed by sonication; lysates were clarified by centrifugation. These lysates were subjected to Strep-tactin® Superflow® affinity chromatography columns (IBA) pre-equilibrated with buffer A for further purification. The column was washed with two column volumes of the same buffer to remove unbound proteins. Proteins were eluted with 2.5 mM desthiobiotin in buffer A and analysed on SDS-PAGE. The fractions containing proteins (End and XthA) were pooled separately and dialyzed against 20 mM Tris-HCl (pH 8.0). Further purification of End was carried out by anion exchange chromatography by using Resource Q (GE Healthcare) pre-equilibrated with 20 mM Tris-HCl (pH 8.0), on an AKTA FPLC purifier (GE Healthcare). End was eluted from the Resource Q column by using a linear gradient of 0 to 0.5 M NaCl in the same buffer; protein eluted at 100 mM NaCl and was dialyzed against 20 mM Tris-HCl (pH 8.0) prior to storage. XthA was purified by using gel filtration chromatography by using Superdex 200 (GE Healthcare) column, pre-equilibrated with 20 mM Tris-HCl (pH 8.0). The purity of each protein was analyzed by SDS–PAGE on a 12.5% polyacrylamide gel. Purified proteins were collected and stored as aliquots at −80°C till further use.

### DNA Substrates

Oligonucleotides (Table 1 and Table S1) were purchased from Sigma except the oligonucleotide containing a central synthetic (tetrahydrofuranyl, THF) AP site that was purchased from Glen research.

**Table 1:** DNA substrates employed in the study.

Oligo number	Name of oligomers	Sequence (5′→ 3′)	Length (nt)	Oligomer no. annealed for DS substrate	Name of DS substrate
**Oligonucleotides for AP endonuclease activity**					
1	subSS-19AP	*ggcgaacgagacgagggc(**AP**)gctggaaagg	29		
2	subSS-19AP(T)	cctttccagc**T**gccctcgtctcgttcgcc	29	1and 2	subAP·T/subDS-AP
3	subSS-19AP(A)	cctttccagc**A**gccctcgtctcgttcgcc	29	1and 3	subAP·A
4	subSS-19AP(G)	cctttccagc**G**gccctcgtctcgttcgcc	29	1and 4	subAP·G
5	subSS-19AP(C)	cctttccagc**C**gccctcgtctcgttcgcc	29	1and 5	subAP·C
**Oligonucleotides for 3**′**→5**′ **exonuclease activity**					
6	subSS-Exo	*gggactctcgaggaatgcg	19		
7	subSS-Rec	ggcgcattcctcgagagtccc	21	6 and 7	subDS-REC
8	subSS-Blunt	cgcattcctcgagagtccc	19	6 and 8	subDS-BLUNT
9	subSS-Nick1	cagctaatggctagcggc	18		
10	subSS-Nick2	gccgctagccattagctgcgcattcctcgagagtccc	37	6, 9 and 10	subDS-NICK

SS is single-strand; DS is double-strand; nt is nucleotides; AP is the abasic site; asteriks (*) denotes the γ-^32^P radioactively labelled terminus.

The substrates employed in the enzymatic assays are mentioned in Table 1. 5′ end labeling of the oligonucleotides was performed in a 50 µl reaction at 37°C for 30 min by using T4 polynucleotide kinase (New England Biolabs) and [γ-^32^P] ATP (3000 Ci/mmol) (BRIT, India) in the kinase buffer supplied by the manufacturer. Free nucleotides were then removed from the reaction mix by using QIAquick® Nucleotide Removal Kit (QIAGEN) and the oligo was eluted in nuclease-free water (QIAGEN). The 5′ end-labeled oligonucleotides were annealed in equimolar quantities to their appropriate complementary oligonucleotides in a buffer containing 50 mM NaCl and 10 mM Tris-HCl (pH 8.0) by heating to 95°C for 5 min followed by cooling to room temperature as described previously [35]. The single-stranded oligonucleotide substrate bearing the synthetic AP site, tetrahydrofuranyl (AP) was designated as subSS-19AP. The double-stranded form of the substrate, was generated by annealing subSS-19AP with complementary strand subSS-19AP(T), subSS-19AP(A), subSS-19AP(G) or subSS-19AP(C) where either thymine, adenine, guanine or cytosine was paired opposite to the AP site; the resulting duplex oligonucleotides were referred to as subAP·T (A, G, C), respectively. Routinely, subAP·T was used as substrate for AP endonuclease assays and designated as subDS-AP. To perform 3′→5′ exonuclease assays, subSS-Exo was employed as the single-stranded substrate. To generate duplex substrates comprising of a 3′ recessed or blunt terminus, subSS-Exo was separately annealed to subSS-Rec or subSS-Blunt to generate subDS-REC or subDS-BLUNT, respectively. A duplex substrate comprising of a nick (subDS-NICK) was also generated by annealing the oligonucleotides subSS-Nick1 and subSS-Nick2 with subSS-Exo.

### Enzymatic Assays

The AP endonuclease assays were performed by using 800 fmol of the 5′ end-labeled substrate and indicated amount of purified protein (either End or XthA) in a standard buffer comprising of 10 mM Tris-HCl (pH 8.0) and 100 µg/ml bovine serum albumin (BSA) in a total volume of 10 µl. For the exonuclease activity of both proteins, 20 fmol of the 5′ end-labeled substrate was employed in standard buffer (same as above) in a volume of 10 µl. To evaluate the exonuclease activity of XthA, the standard buffer was supplemented with 10 mM MgCl_2_. All reactions were carried out for 15 min at 37°C unless stated otherwise. All assays were carried out in duplicates.

The reactions were stopped by transferring tubes to ice and adding an equal volume of loading solution (98% formamide, 10 mM EDTA, 0.5% xylene cyanole and 0.5% bromphenol blue) [36]. The products of the reactions were resolved by denaturing gel electrophoresis (20% polyacrylamide, 8 M urea). The gels were dried and visualized by using a phosphorimager (model FLA-9000, FUJIFILM Corporation, Minato-ku, Tokyo, Japan) and quantification of the results was carried out by using the Multi Gauge V3.0 software (FUJIFILM Corporation, Minato-ku, Tokyo, Japan). To eliminate the error due to unequal loading, if any, the concentration of the reaction products were calculated from the ratio of the intensity of bands of products to the intensity of the total radioactivity in each lane. Graphs were generated by using Prism 5 software (Version 5.01; GraphPad Software Inc., CA, USA).

### AP Endonuclease Assay by Employing Mycobacterial Cell-free Protein Extracts

The cultures of parental and mutant (lacking either End or XthA or both) mycobacterial strains were grown to an A_600 nm_ of 0.8 in MB7H9 medium at 37°C in an orbital shaker at 200 rpm. 50 ml culture was harvested by centrifugation, washed once with equal volume of phosphate buffered saline (PBS), pH 7.4. Cells were resuspended in 750 µl of above buffer containing 1 mM PMSF and 1.6 g of 0.1 mm zirconium beads were added. Cells were lysed by bead beating at 5000 vibrations per minute for 8 times with 30 second pulses. Lysates were centrifuged at 12000 rpm for 20 min at 4°C. The cell-free protein extract was filtered through 0.2 µm filter followed by sonication for 2 min. The amount of protein in the lysate was quantified by using Bradford’s method. The lysate was used for AP endonuclease assay in a 10 µl reaction volume by employing 20 fmol of 5′ end-labeled substrate (subDS-AP), 1 µg of *M.tuberculosis* lysates, 100 µg/ml BSA, 2 mM MgCl_2_ in 10 mM Tris-HCl (pH 8.0). Reactions were carried out at 37°C for 30 min.

### Disk Diffusion Assay

The parental and mutant strains (lacking either End or XthA or both) were exposed to oxidative stress inducing agent cumene hydroperoxide (CHP) by employing disk diffusion assay as described previously [37]. Briefly, all the mycobacterial strains were grown to an A_600 nm_ of 0.8 in MB7H9 supplemented medium. The cells were washed once with equal volume of PBS (pH 7.4) followed by dilution to an A_600 nm_ of 0.5 and 200 µl (2×10^6^ cells) was plated on MB7H11 agar supplemented with 10% OADC. The plates were allowed to dry following which a 5 mm sterile disk was placed on the plate. CHP was added at 5 mM and 10 mM concentrations on to the disk (in a 10 µl volume). DMSO was used as the solvent control. After incubation for 2 weeks at 37°C, the growth inhibition zone was measured.

## Results

### Purification and AP Endonuclease Activity of End and XthA

**Figure 1:**
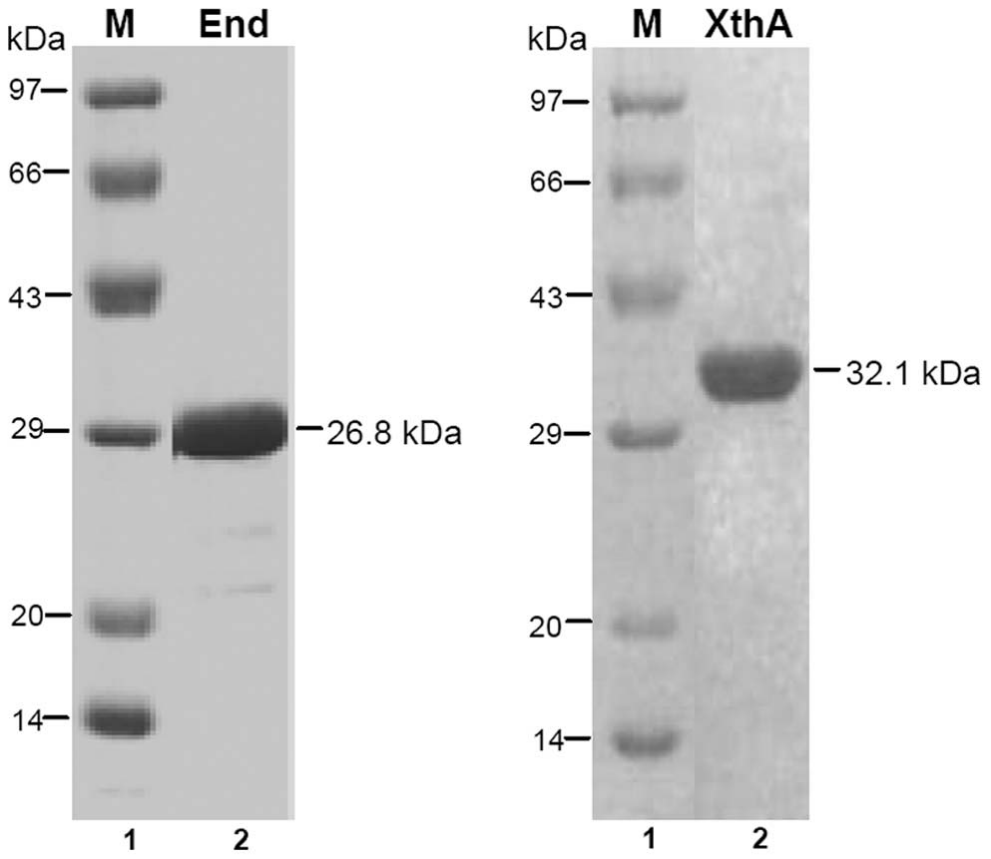
Analysis of expression of End and XthA by SDS - PAGE using a 12.5% gel. Purified proteins End (26.8 kDa) and XthA (32.1 kDa) were analyzed on a 12.5% SDS PAGE. Lane 1, protein molecular weight marker; lane 2, 2.5 µg of purified protein.

The cloning of *end* and *xthA* in pet-21c(+) vector was confirmed by restriction digestion (data not shown). The constructs were verified for the absence of errors by sequencing of plasmid inserts. End (26.8 kDa) and XthA (32.1 kDa) were purified as described in materials and methods. Analysis of the purified fractions by SDS-PAGE and Coomassie Blue staining showed that the End and XthA proteins were >95% pure (Fig. 1). These proteins were assayed for AP endonuclease activity.

**Figure 2:**
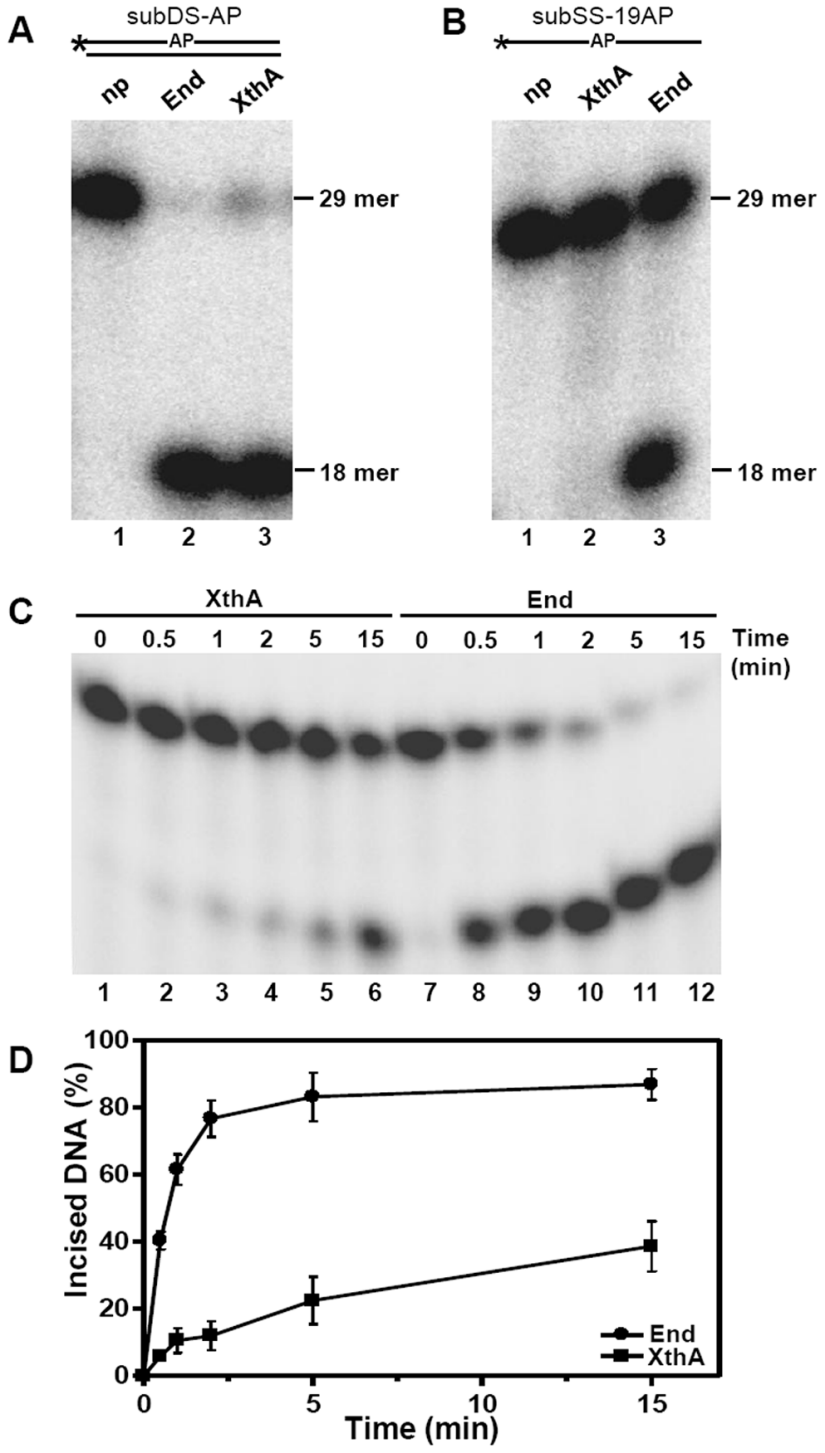
End displays stronger AP endonuclease activity in comparison to XthA. Schematic diagram of oligonucleotide substrates in which asterisk (*) and AP denote the radioactively labeled γ-^32^P terminus and the abasic site in the substrate, respectively. 5′ end-labeled substrate (200 fmol) was incubated with 5 fmol of either End or XthA at 37°C. (A) Lanes 1–3, subDS-AP was incubated without protein (np), with End or with XthA, respectively, for 30 min. (B) Lanes 1–3, subSS-AP was incubated without protein, with XthA or with End, respectively, for 30 min. (C) Time dependence of End and XthA on AP endonuclease activity. The reaction was carried out for different intervals of time (0–15 min). The products were analyzed by denaturing PAGE on 20% polyacrylamide gels containing 8 M Urea, and the DNA bands were visualized by autoradiography. The position of 29 mer substrate and 18 mer incision product is indicated. (D) Graphical representation of results in C. The data in the graph represent mean (±standard errors) of two independent experiments carried out in duplicates.

The natural substrate for AP endonuclease enzyme is an AP site within a double-stranded DNA. Initially, to determine whether the *M.tuberculosis* AP endonucleases were active at the AP site, a 5′ end-labeled double-stranded 29-nt substrate (subDS-AP,100 fmol), in which one strand contains a central synthetic AP site, tetrahydrofuranyl (THF), at position 19 was incubated for 30 minutes separately with 10 fmol of either purified End or XthA as described in materials and methods. Class II AP endonuclease would characteristically result in cleavage at the AP site in this substrate, generating two primary cleavage products: an end-labeled 18-nt fragment and an unlabelled 11-nt fragment. Incubation of the 29-nt duplex DNA substrate with the *M.tuberculosis* AP endonucleases generated an 18-nt labeled oligomer indicating that End and XthA catalyze the hydrolysis of 5′phosphodiester bond upstream to an abasic site. Significant AP endonuclease activity was observed for each of these proteins (Fig. 2A, lanes 2 and 3). End and XthA cleaved 92% and 71% of the substrate, respectively, after 30 minutes of incubation. However, when these proteins were tested for AP endonuclease activity against the single-strand DNA substrate (subSS-19AP), the activity was observed only for End (Fig. 2B, lane 3) but not for XthA (Fig. 2B, lane 2). Hence, End endonucleolytically cleaves both single-strand and double-strand AP substrate, while XthA acts only on double-strand AP substrate. In addition, it was observed that the AP endonuclease activity of the End enzyme was ∼2 fold higher for double-stranded DNA in comparison to single-stranded DNA as quantified by the level of cleavage at the AP site in the substrates. The high degree of purity of both the proteins strongly suggested that the observed AP endonuclease activity was intrinsic to the *M.tuberculosis* AP endonucleases.

Examination of activity profiles for these enzymes revealed that the kinetics for both enzymes were significantly dissimilar, with End exhibiting a much faster rate of conversion of the substrate (subDS-AP) to the product in comparison to XthA (Fig. 2C and Fig. 2D). 40% of the substrate was cleaved by End within 30 sec of incubation, while XthA exhibited an incision approximating only to 5%. After 5 min of incubation, the incision of the substrate by End and XthA increased to ∼80% and 20%, respectively. Hence, AP endonuclease activity of End was observed to be 3–4 fold higher than XthA. Moreover, the reaction employing End proceeded to near completion within 15 min whereas, XthA exhibited only 38% incision activity of the substrate in the same time substantiating the higher efficiency of End in comparison to XthA. We calculated the kinetic parameters for these enzymes by employing substrate subDS-AP. The V_max_ and K_cat_ values of the End were estimated to be 41.31±2.44 pmoles/min/µg and 0.0193 sec^−1^, respectively, while for XthA these were estimated to be 10.39±0.86 pmoles/min/µg and 0.0055 sec^−1^. End and XthA displayed classical Michaelis-Menten kinetics for the substrate subDS-AP with K_m_ values of 6.91±1.24 nM and 7.52±2.35 nM, respectively. Moreover, the specificity constant (K_cat_/K_m_) for End (2.79 µM^−1^sec^−1^) was four fold higher than for XthA (0.74 µM^−1^sec^−1^). From the above results we hypothesize that *M.tuberculosi*s End, which displays a higher efficiency in comparison to XthA may be playing a more crucial role in the repair of damaged DNA comprising of AP sites.

### Effect of Metal Ions and Reducing Conditions on the AP Endonuclease Activity of *M.tuberculosis* End and XthA

**Figure 3:**
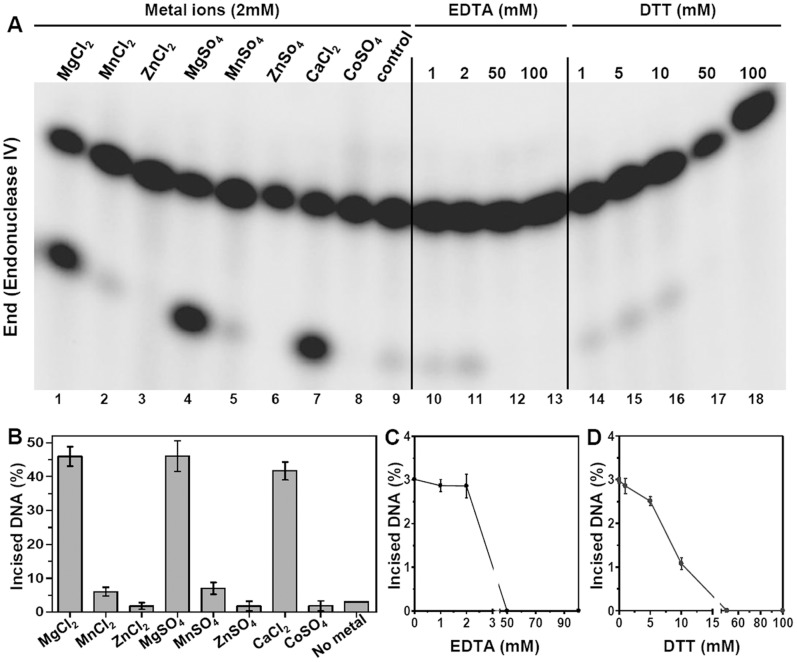
Endonuclease IV is stimulated by the addition of Magnesium and Calcium ions. The 5′ end-labeled substrate subDS-AP (800 fmol) was incubated with 2 fmol of End with various metal ions at a concentration of 2 mM (lanes 1–8), with EDTA (lanes 10–13) and with DTT (lanes 14–18). Concentrations (mM) of EDTA and DTT are indicated on the figures. Substrate incubated with the enzyme without the addition of metal ion, EDTA or DTT was used as control (lane 9). Graphical representation of lanes 1–9, 10–13 and 14–18 of figure A is shown in B, C and D, respectively. The data in the graphs represent mean (±standard errors) of two independent experiments carried out in duplicates. The reaction was carried out for 15 min at 37°C and the products were analyzed on 20% polyacrylamide gels containing 8 M Urea and the DNA bands were visualized by autoradiography.

To evaluate the effect of different metal ions on the AP endonuclease activity of End and XthA, 2 fmol of each protein was separately incubated with 800 fmol of 5′ end-labeled AP substrate (subDS-AP) and reactions were carried out in the presence and absence of various metal ions. End cleaved ∼3% of the AP sites in the substrate in the absence of metal ions, whereas in the presence of MgCl_2_, MnCl_2_, ZnCl_2_, MgSO_4_, MnSO_4_, ZnSO_4_, CaCl_2_ and CoSO_4_, the cleavage was ∼46, 6, 3, 46, 7, 0.7, 41 and 0.7%, respectively (Fig. 3A, lanes 1–9 and Fig. 3B). Hence, it was apparent that the AP endonuclease activity of End was maximally stimulated in the presence of Mg^2+^ (Fig. 3A, lanes 1 and 4) and Ca^2+^ (Fig. 3A, lane 7), both leading to an approximately 15 fold increase in AP endonuclease activity in comparison to the reaction without addition of metal (Fig. 3A, lane 9). End displayed a slight (∼2-fold) increase in its AP endonuclease activity on the substrate when incubated with Mn^2+^ (Fig. 3A, lanes 2 and 5) while no change in activity was observed in the presence of Zn^2+^ or Co^2+^ (Fig. 3A, lane 3, 6 and 8) in comparison to the activity without addition of any metal.

**Figure 4:**
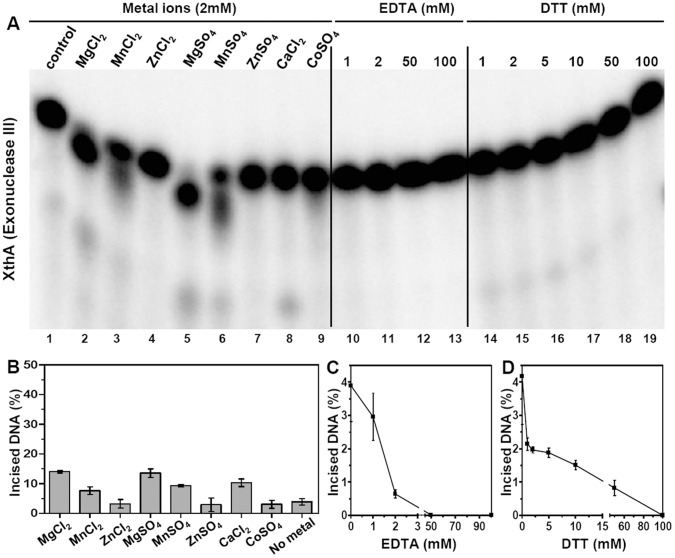
Exonuclease III is stimulated by the addition of Magnesium and Calcium ions. The 5′ end-labeled substrate subDS-AP (800 fmol) was incubated with 2 fmol of XthA with various metal ions at a concentration of 2 mM (lanes 2–9), with EDTA (lanes 10–13) and with DTT (lanes 14–19). Concentrations (mM) of EDTA and DTT are indicated on the figures. Substrate incubated with the enzyme without the addition of metal ion, EDTA or DTT was used as control (lane 1). Graphical representation of lanes 1–9, 10–13 and 14–19 of figure A is shown in B, C and D, respectively. The data in the graphs represent mean (±standard errors) of two independent experiments carried out in duplicates. The reaction was carried out for 15 min at 37°C and the products were analyzed on 20% polyacrylamide gels containing 8 M Urea and the DNA bands were visualized by autoradiography.

Similarly, the effect of different metal ions on the AP endonuclease activity of XthA was examined (Fig. 4A, lanes 1–9 and Fig. 4B). In the absence of any added metal, XthA cleaved ∼4% of the AP sites in the subDS-19AP substrate. In the presence of MgCl_2_, MnCl_2_, ZnCl_2_, MgSO_4_, MnSO_4_, ZnSO_4_, CaCl_2_ and CoSO_4_, the cleavage was ∼14, 8, 3, 14, 9, 4, 10 and 4%, respectively. Similar to the results observed for End, the AP endonuclease activity of XthA was also maximal in the presence of Mg^2+^ (Fig. 4A, lanes 2 and 5). Though, the maximal activity achieved by XthA, in the presence of Mg^2+^ under the standard reaction conditions was observed to be ∼3.5 fold higher than the activity achieved without any metal ion (Fig. 4A, lane 1), it corresponded to only 14% of the cleaved product, as against 46% product observed in the case of End on addition of this metal. The activity of XthA observed in the presence of Mn^2+^ (Fig. 4A, lanes 3 and 6) or Ca^2+^ (Fig. 4A, lane 8) was comparable resulting in ∼2 fold increase in AP endonuclease activity while addition of Zn^2+^ or Co^2+^ resulted in no significant enhancement of AP endonuclease activity (Fig. 4A, lanes 4, 7 and 9). Interestingly, it was observed during this study that in the presence of Magnesium and Manganese, XthA cleaved the 29-nt substrate to not only 18-nt primary cleavage product, but to products that appeared ∼1–2 nucleotides smaller than the 29-nt substrate as well, indicating the possibility of XthA exhibiting an 3′→5′ exonuclease activity in addition to the AP endonuclease activity. For this reason, it appeared that these metals are important for both AP endonuclease and exonuclease activity of XthA.

The AP endonuclease activity of both the proteins was tested for their sensitivity to the metal chelator, EDTA (Fig. 3A and 4A, lanes 10–13). End was tolerant to EDTA upto a concentration of 2 mM (Fig. 3C) while XthA was highly sensitive to increasing concentrations of EDTA and a significant reduction in AP endonuclease activity was observed (Fig. 4C). The activity was abolished completely at 50 mM EDTA in case of both the proteins. This result again emphasized the importance of metal ions in the AP endonuclease activity of both these mycobacterial proteins.

The effect of the reducing agent DTT on the AP endonuclease activity of End (Fig. 3A, lanes 14–18 and Fig. 3D) and XthA (Fig. 4A, lanes 14–19 and Fig. 4D) was investigated. Addition of increasing concentrations of DTT to the reaction mixture reduced the AP endonuclease activity of both the enzymes. The complete inhibition of AP endonuclease activity of End and XthA was seen at DTT concentrations of 50 mM and 100 mM, respectively. These results indicate that the AP endonucleases may be more active under oxidative rather than reductive environments.

### Base Pair Specificity of End and XthA during AP Endonuclease Activity

**Figure 5:**
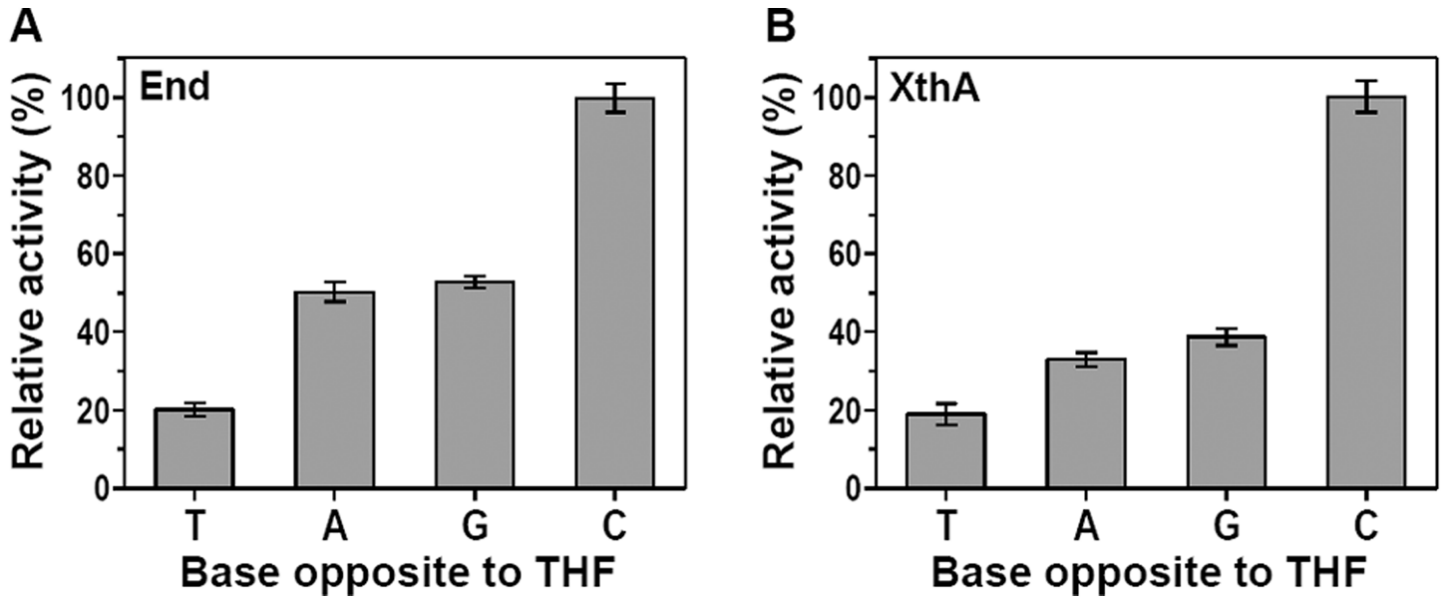
Preferential recognition of cytosine opposite the abasic site by End and XthA. Each of the four 5′ end-labeled substrates subAP·T/A/G/C (800 fmol) were separately incubated with 2 fmol of either End (A) or XthA (B) for 25 min at 37°C. The AP endonuclease activity was measured under standard assay conditions and plotted as relative activities when acting on different duplex substrates. The data represents mean (±standard errors) of two independent experiments carried out in duplicates.

To examine the influence of the base opposite to an AP site on the incision activity, we assayed the *M.tuberculosis* AP endonucleases. The base pair specificity of End and XthA was investigated by using 2 fmol of enzyme and 800 fmol of the duplex oligonucleotides (subAP·T, subAP·A, subAP·G or subAP·C) containing each of the four naturally occurring bases opposite the AP site. The incision activity was measured for each enzyme on the DNA substrates as relative activity of the enzyme when acting upon the four different duplexes (Fig. 5). The repair of AP residues exhibited a marked preference for the opposite base. End (Fig. 5A) and XthA (Fig. 5B) both strongly preferred an AP site paired opposite to cytosine (AP·C), exhibiting a 5 fold higher incision efficiency in comparison to AP·T mismatch. AP·T was incised least efficiently by either enzyme. AP·A and AP·G mismatches were incised with quite similar efficiency in DNA substrates by individual enzymes. These results demonstrate that the incision activity of *M.tuberculosis* AP endonucleases is influenced by the base that is paired opposite to the AP site, displaying a marked preference for Cytosine residue.

### 3′→5′ Exonuclease Activity of *M.tuberculosis* AP Endonucleases

**Figure 6:**
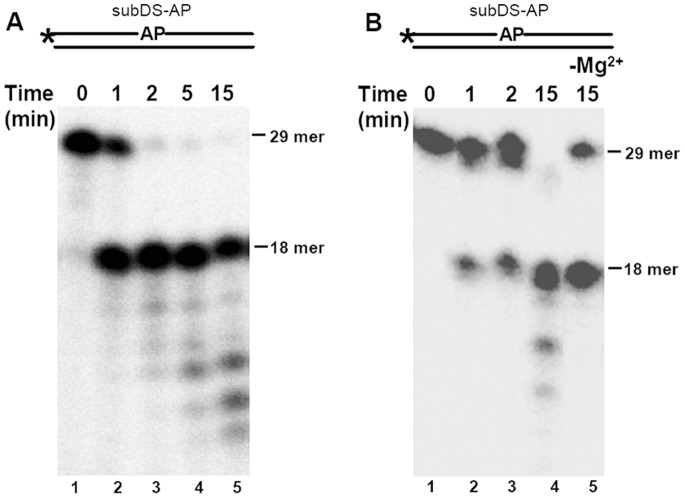
3′→5′ exonuclease activity of End and XthA on AP substrate. Schematic diagram of oligonucleotide substrates in which asterisk (*) and AP denote the radioactively labeled γ-^32^P terminus and the abasic site in the substrate, respectively. 5′ end-labeled substrate subDS-AP (20 fmol) was incubated with 10 fmol of either End (A) or XthA (B) for variable time at 37°C. (A) Lanes 1–5, incubation of substrate with End in standard buffer for 0, 1, 2, 5 and 15 min, respectively. (B) Lanes 1–4, incubation of substrate with XthA in standard buffer containing 10 mM MgCl_2_ for 0, 1, 2 and 15 min, respectively. Lane 5, XthA was incubated with the substrate for 15 min in standard buffer without Magnesium. The position of 29 mer substrate and 18 mer incision product is indicated.

We next evaluated the exonuclease activity of the *M.tuberculosis* AP endonucleases by using subDS-AP as the substrate. However, a higher amount of enzyme to substrate ratio was used for the characterization to facilitate examination of the effect of enzyme on the activity and the extent to which the enzyme progressed on the substrate. 20 fmol of the substrate was incubated with 10 fmol of either End (Fig. 6A) or XthA (Fig. 6B) for different time intervals. Under these reaction conditions, besides the endonucleolytic cleavage at the AP site, a significant 3′→5′ exonuclease activity of the substrate was observed in the case of each enzyme. The exonuclease activity was observed after 2 min and 15 min of incubation of the substrate with End and XthA, respectively. Moreover, the exonuclease activity of End did not require any additional metal ion (data not shown). In contrast, XthA was highly dependent on metal ion for its AP endonuclease and 3′→5′ exonuclease activities. The AP endonuclease activity of XthA reduced by 29% in the absence of metal ions (Fig. 6B, lane 5) in comparison to its activity in the presence of metal ion (Fig. 6B, lane 4). More importantly, a complete abrogation of the exonuclease activity was observed in the absence ofmetal ions. Hence, XthA is dependent on the metal ions for complete manifestation of both the activities.

**Figure 7:**
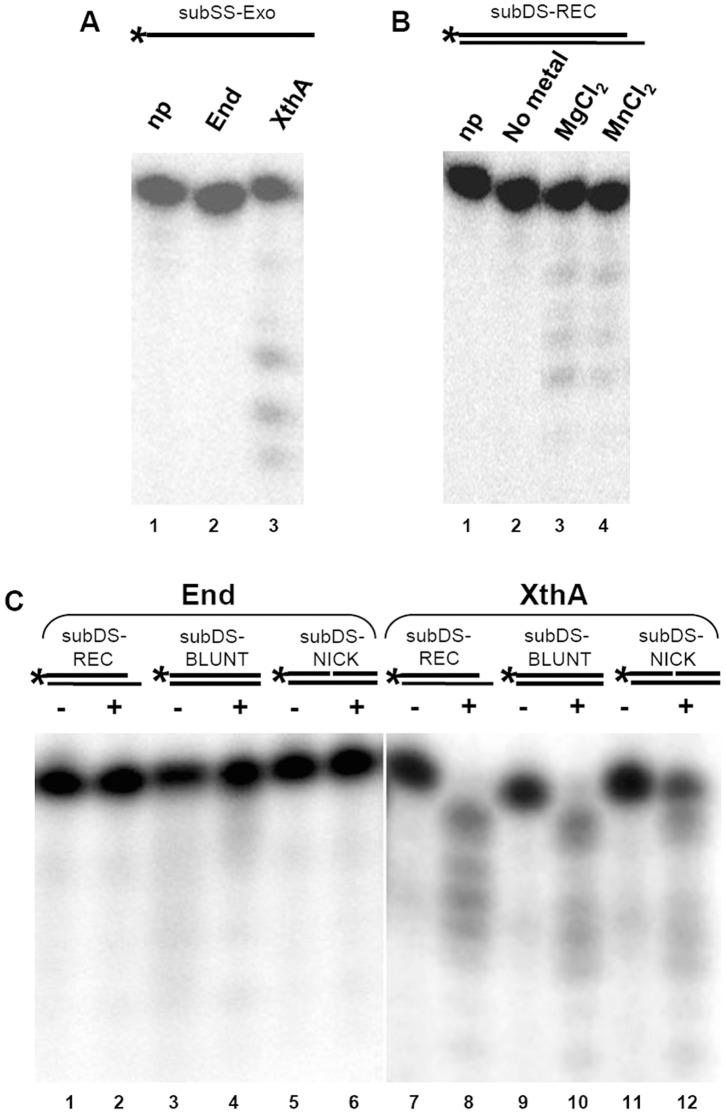
XthA displays a metal ion dependent 3′→5′ exonuclease activity on a large variety of substrates. Schematic diagram of oligonucleotide substrates (refer Table 1 for details) in which asterisk (*) denotes the radioactively labeled γ-^32^P terminus. (A) 20 fmol of 5′ end-labeled substrate subSS-Exo was incubated without protein (np,lane 1), with 2 fmol of either End (lane 2) or XthA (lane 3) in a buffer containing 10 mM MgCl_2_ for 15 min at 37°C. (B) 20 fmol of 5′ end-labeled substrate subDS-REC (3′ recessed substrate) was incubated without protein (lane 1), with 2 fmol of XthA without any metal (lane 2), XthA in a buffer containing 10 mM MgCl_2_ (lane 3) or XthA in a buffer containing 10 mM MnCl_2_ (lane 4). (C) 20 fmol of each of the 5′ end-labeled DNA substrates (subDS-REC, subDS-BLUNT and subDS-NICK) were incubated in the absence (–) or presence (+) of 10 fmol of End (lanes 1–6) and XthA (lanes 7–12).

AP endonucleases have been reported to be most active on 3′ recessed DNA substrates in comparison to other substrates for the display of exonuclease activity [38], [39]. Hence, to study the exonuclease activity of mycobacterial AP endonucleases a similar substrate was generated by annealing a 5′ end-labeled 19-nt oligomer (subSS-Exo) with a 21-nt oligomer (subSS-Rec) to generate a double-stranded exonuclease substrate subDS-REC (3′ recessed substrate). End did not display exonuclease activity when incubated with either single-stranded (subSS-Exo, Fig. 7A, lane 2) or double-stranded substrate (subDS-REC, Fig. 7C, lanes 1 and 2). On the other hand, it was observed that incubation of XthA with either a 5′ end-labeled single-strand oligomer, subSS-Exo (Fig. 7A, lane 3), or with a double-strand 3′ recessed oligomer, subDS-REC (Fig. 7B, lanes 3 and 4), resulted in the gradual shortening of the 19 mer template that indicated an exonucleolytic digestion of the DNA from the 3′ end. Examination of exonuclease activity on the subDS-Rec by employing XthA, demonstrated the exonuclease digestion of the substrate both in the presence of Mg^2+^ or Mn^2+^ ions which was abolished in the absence of metal ions (Fig. 7B, lane 2). Hence, the exonuclease activity of XthA was strongly dependent upon metal ion. To further determine the DNA substrate specificity, we compared the exonuclease activity of End and XthA for their ability to hydrolyze DNA duplexes by employing substrates with either a blunt (subDS-BLUNT) or a recessed 3′-terminus (subDS-REC) or by using a substrate containing an internal nick (subDS-NICK) in the heteroduplex DNA. End displayed no activity on any of the above substrates (Fig. 7C, lanes 1–6). In contrast, XthA exhibited strong exonuclease activity on a recessed 3′ end containing partial DNA duplex, with 10% uncleaved substrate (Fig. 7C, lane 8) and on a blunt-end containing heteroduplex DNA, with 14% uncleaved substrate (Fig. 7C, lane 10). In both the above cases, more than 85% of the substrate was cleaved further to multiple bands smaller in size than the labeled 19-nt fragment indicating the presence of exonuclease activity. Though, the nicked substrate was cleaved and the band sizes decreased approximately one base at a time, XthA showed weaker activity on a nicked DNA in comparison to recessed and blunt ended substrates (Fig. 7C, lane 12) displaying ∼35% of uncleaved subDS-NICK.

### AP Endonuclease Activity in Cell-free Protein Extracts of *M.tuberculosis*

**Figure 8:**
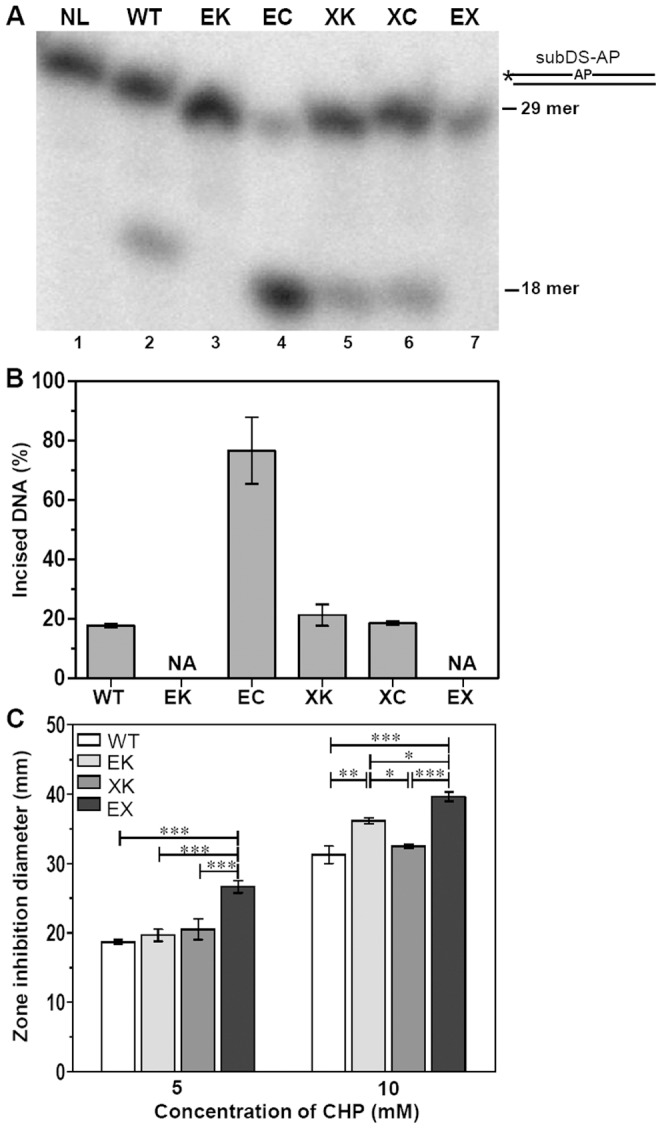
End is the major AP endonuclease in the mycobacterial cell-free protein extracts. (A) Schematic diagram of oligonucleotide substrate in which asterisk (*) and AP denote the radioactively labeled γ-^32^P terminus and the abasic site in the substrate, respectively. The 5′ end-labeled substrate subDS-AP (20 fmol) was incubated with cell-free protein extracts (1 µg) of parental and mutant strains of *M.tuberculosis* for 30 min at 37°C. (B) The graph represents mean (±standard errors) of three independent experiments, of which one is shown in (A). (C) Influence of disruption of single or both the AP endonucleases on the ability of *M.tuberculosis* to withstand oxidative stress generated by cumene hydroperoxide (CHP). Graph depicts the range of the inhibition zones observed in the presence of various concentrations of cumene hydroperoxide. The values are represented as the mean (± standard errors) of two independent experiments carried out in triplicates. ∗,*P*<0.05; ∗∗,*P*<0.01; ∗∗∗,*P*<0.001 (Two way ANOVA). NL, no lysates; NA, no activity; WT, *M.tuberculosis*; EK, MtbΔ*end*; EC, MtbΔ*end*Comp; XK, MtbΔ*xthA*; XC, MtbΔ*xthA*Comp; EX, MtbΔ*end*Δ*xthA*.

To understand the specific role(s) of these AP endonucleases in *M.tuberculosis*, we generated single (MtbΔ*end* and MtbΔ*xthA*) and double (MtbΔ*end*Δ*xthA*) mutants (Puri, R.V. *et. al*, unpublished data) by employing recombineering method [34]. Cell-free protein extracts of the parental and mutant strains were prepared and examined for AP endonuclease activity (Fig. 8A and Fig. 8B) as described in materials and methods. Incubation of the 29-nt duplex DNA substrate (subDS-AP) with the *M.tuberculosis* parental cell-free extract generated a 18-nt labeled oligomer (17% incised substrate, Fig. 8A, lane 2) indicating that AP endonucleases catalyze the hydrolysis of the 5′ phosphodiester bond upstream to the abasic site. On incubation of this substrate with MtbΔ*end*, a complete abrogation of AP endonuclease activity was observed (Fig. 8A, lane 3). However, this activity was restored when the *end* gene was complemented on a plasmid and was in fact, 4 fold higher than the parental strain (76% incised substrate, Fig. 8A, lane 4). This increased activity of MtbΔ*end*Comp may be attributed to the higher copy number of the plasmid used for the complementation [40], [41], [42]. The activity was comparable in the parental and MtbΔ*xthA* (20% incised substrate) implying that XthA did not contribute to the AP endonuclease activity in the mycobacterial cells under the experimental conditions. The activity of MtbΔ*xthA*Comp was similar to the parental strain (18% incised substrate). These results indicate that under normal physiological conditions, most of the AP endonuclease activity in *M.tuberculosis* is represented by End.

Further, to evaluate the role of AP endonucleases in the protection of *M.tuberculosis* against the DNA damages caused by oxidative radicals, the toxic oxidant cumene hydroperoxide (CHP) was employed. CHP, an alkylperoxide, is a stable free radical source produced inside the cells by metabolism of unsaturated fatty acids and nucleic acids [43]. Figure 8C depicts that the MtbΔ*end*Δ*xthA* double mutant exhibited an enhanced sensitivity to CHP as demonstrated by larger zones of inhibition relative to either parental or the single mutant strains in the disc diffusion assay. At low concentration of CHP (5 mM), both MtbΔ*end* and MtbΔ*xthA* displayed zones of inhibition similar to the parental strain. However, as the level of oxidative damage increased (10 mM CHP), the absence of End (MtbΔ*end* mutant) resulted in a significant increase in the inhibition zone in comparison to the parental strain, while mutation in *xthA* alone (MtbΔ*xthA* mutant) did not result in any enhancement in the sensitivity to CHP. Our results suggest that in *M.tuberculosis*, the major role of protecting the DNA against oxidative damage is played by End. These observations are in agreement with our hypothesis that End, the more efficient AP endonuclease, is of higher significance to *M.tuberculosis* in the repair of damaged DNA by BER pathway.

## Discussion

*M.tuberculosis* encounters the assaults on all its biomolecules, including DNA, that are inflicted upon by the reactive oxygen and nitrogen species produced as a part of the host’s innate immune response. In its struggle to survive in the host, this pathogen must possess efficient DNA repair mechanisms to safeguard its genetic machinery. AP endonucleases are a class of enzymes responsible for the removal of abasic sites in DNA, which are generated in response to spontaneous hydrolysis or modifications of the bases. However, there have been no earlier reports on the biochemical characterization of AP endonucleases from this important pathogen [6]. Based on the sequence analysis of these AP endonuclease genes in clinical strains of *M.tuberculosis*, it has been demonstrated that *end* displayed variations in sequence in certain clinical strains and in several other strains a single nucleotide deletion was found in this gene [44]. On the other hand, among the 100 strains evaluated in this study, *xthA* was amongst the few genes that displayed no sequence variation or deletion indicating thereby that *xthA* may play a more important role in *M.tuberculosis* than *end* [44]. However, the experimental evidence gathered by us in the current study, has made interesting revelations in support of End being the more important AP endonuclease of *M.tuberculosis*. We have evaluated the enzymatic activities of the annotated AP endonucleases of *M.tuberculosis* [33], and show that both Endonuclease IV (End) and Exonuclease III (XthA) are multifunctional enzymes. These enzymes exhibit AP endonuclease and 3′→5′ exonuclease activities with differences in their substrate specificities. More importantly, we show that Endonuclease IV is the major AP endonuclease of *M.tuberculosis* that also plays an important role in protecting the pathogen against oxidative DNA damage.

Previous studies have indicated that sequence of the DNA substrate may affect the excision activity of enzymes [45], [46], [47], [48]. We have utilized well characterized oligonucleotide substrates that have been employed by several groups for routine BER assays [36], [38]. Evaluation of *M.tuberculosis* AP endonucleases revealed that End is a highly efficient AP endonuclease while XthA displays weaker AP endonuclease activity. This difference in the efficiency of End is attributed to a ∼4 fold higher K_cat_ value of End in comparison to XthA when acting on a double-stranded DNA containing an AP site. Mycobacterial End cleaved both the double and single-stranded DNA containing an AP site. This finding is similar to the endonuclease IV of *Chlamydia pneumoniae* (CpEndoIV), an obligate intracellular bacterium that infects humans and is a major cause of pneumonia [49]. Quantification of the level of cleavage at the AP site in the substrates showed that the AP endonuclease activity of the End enzyme exhibited almost 2-fold higher AP endonuclease activity on double-stranded DNA in comparison to single-stranded DNA; a finding also reported for Ec-EndoIV [36]. Though the significance of excision of AP sites from single-stranded DNA substrates has been a matter of debate, a number of phylogenetically diverse DNA glycosylases in BER have shown activity against damaged bases in single-stranded DNA suggesting that BER may at least be initiated in single-stranded DNA [50], [51], [52], [53]. The DNA glycosylase activity directed against a modified base in single-stranded DNA would generate an AP site in the single-stranded DNA. Besides, the incision activity on AP site in single-stranded DNA has also been reported for other AP endonucleases such as CpEndoIV, Ec-EndoIV and human APE1 [36], [49], [54].The above results promote the understanding of mechanism of AP site recognition by endonuclease IV and are suggestive of a probable role of End in the repair of AP site of single-stranded DNA during replication and/or transcription *in vivo* apart from its major function of AP site removal from the double-stranded DNA. However, the cleavage activity of mycobacterial XthA on the single-stranded DNA containing an AP site was not detected.

In *E.coli*, the association of metal ions with Ec-EndoIV and Ec-ExoIII is well established [12], [55], [56] which stimulated us to evaluate the effect of metal ions on the AP endonuclease activity of mycobacterial End and XthA. The primary role of the metal ion is to promote a conformational change in the DNA containing abasic site, priming it for enzyme-mediated hydrolysis [57]. Previous studies have demonstrated the importance of metal ions (Mg^2+^, Ca^2+^, Co^2+^, Zn^2+^ and Mn^2+^) for the activity of AP endonucleases across various species [55], [56], [57], [58], [59], [60], [61], [62]. The sequence alignment of mycobacterial AP endonucleases End and XthA with their homologues in Endonuclease IV and Exonuclease III family, reveals complete conservation of metal binding sites in End (His56, His96, Glu129, Asp162, His165, His191, Asp204, His206 and Glu233) and XthA (Asn32, Glu57, Asp180, Asn182 and His281) [63], [64]. Our results demonstrate that the activity of AP endonucleases of *M.tuberculosis* is stimulated in the presence of Mg^2+^ or Ca^2+^ and these metals may play an important role in the catalysis of these enzymes. Inhibitory effect of EDTA on the AP endonuclease activity of both the proteins further substantiates that like other members of Endonuclease IV and Exonuclease III family, both the mycobacterial AP endonucleases appear to require transition metals for their activity. In the host macrophages, the pathogen encounters oxidative stress that results in a variety of DNA damaging lesions including AP sites. Moreover, there is experimental evidence for an increase in the number of AP sites in DNA after exposure to oxidative stress implicating the role of AP endonucleases under such conditions [65]. Our results demonstrate that the addition of the reducing agent DTT inhibited the progression of the enzyme on the substrate suggesting that these enzymes may be more active when the cell is under oxidative stress substantiating their *in vivo* role.

We next analyzed the influence of the base opposite the AP site (THF) on the nicking activity of End and XthA. The preferential recognition of a modified residue paired opposite to one of the four natural bases in duplex DNA is an important feature of DNA repair enzymes [66]. An oligonucleotide harboring an AP site was annealed with complementary strand with different bases opposite AP site prior to treatment with the enzymes. Both End and XthA were five times as active on a THF residue opposite C as compared to T. End and XthA incised AP·A and AP·G mismatches with a similar efficiency. The data obtained with mycobacterial AP endonucleases acting on THF mismatches in the present study, are in agreement with a previous observation employing *S.cerevisiae* Apn1. Apn1 has been observed to display a 5-fold higher activity on a THF residue opposite C or T than opposite G or A [66]. However, the opposite base had no effect on the AP endonuclease activity of *E.coli* AP endonucleases [67]. Cytosine under normal physiological conditions base pairs with guanine. The preferential recognition of AP site opposite the cytosine residue is an important characteristic of mycobacterial AP endonucleases, probably indicating the importance of AP endonucleases in recognizing the modifications in the guanine base. 7,8-dihydro-8-oxoguanine (8-oxoG) is one of the most common damage resulting from the oxidation of DNA, and failure to replace it with the correct base results in mutations [68]. Moreover, it has been demonstrated that DNA polymerase(s) from mycobacteria display a preference for the incorporation of G opposite the 8-oxoG as opposed to an A in *E. coli*, which further enhances the risk of accumulating guanine modifications like 8-oxoG, in their GC rich DNA [29]. Further, several class II AP endonucleases such as yeast Apn1 and human Ape1 have been demonstrated to remove 3′ incorporated 8-oxoG damaged nucleotide [69], [70]. In light of the above, it is not unlikely that the AP endonucleases of *M.tuberculosis* play a role in the removal of 8-oxoG damaged nucleotide.

**Figure 9:**
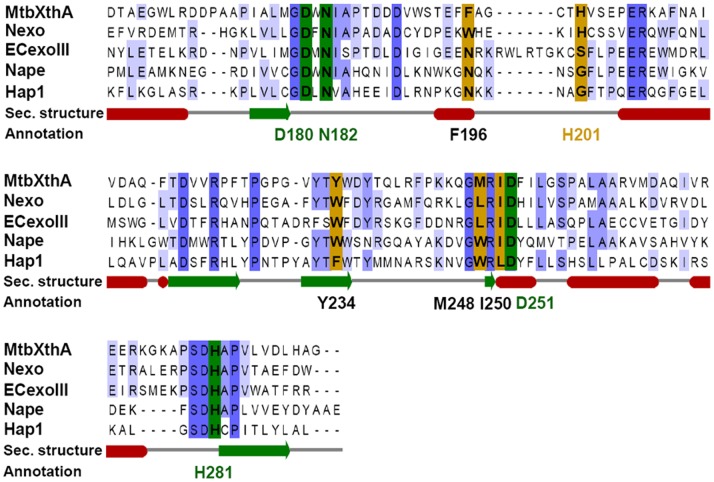
Sequence alignments of XthA with other AP endonuclease homologs. Alignment of the C-terminal 127 residues of MtbXthA (Rv0427c), with non-functional AP endonuclease paralogue of *N.meningitidis* (Nexo) and functional AP endonucleases of *E.coli* (ECExoIII), *N.meningitidis* (Nape) and *Homo sapiens* (Hap1) indicates the plausible role of histidine (H201) for low AP endonuclease activity. The residues involved in abasic ribose binding and catalysis are indicated in brown and green color, respectively. Absolutely conserved residues in the alignment are colored in dark blue and light blue for partial identity. Secondary structure elements are shown in red for α-helices and green for β-strands. CLUSTALW was employed for the initial multiple sequence alignment of proteins [71]. This was followed by using the programme JalView [72] for the prediction of conserved residues and secondary structure.

We observed that XthA is a less efficient enzyme than End for its AP endonuclease activity (Fig. 2C and Fig. 2D). Also, incubation of XthA with metal ions enhanced the AP endonuclease activity of XthA, but to lesser extent than that observed in the case of End (Fig. 3 and Fig. 4). Overall, we observed a lower AP endonuclease activity of XthA when compared to End. Sequence comparison of *M.tuberculosis* XthA, with other well-characterized exonuclease III family AP endonucleases from other species, revealed interesting observations. XthA possesses all the active site residues necessary for AP endonuclease activity (i.e., Asp180, Asn182, Asp251 and His281; Fig. 9). However, XthA comprises of a histidine residue (His201) at the abasic ribose binding pocket, instead of a small amino acid like Glycine or Serine generally observed in functional AP endonucleases (Gly231 in human AP endonuclease, HAP1; Gly170 in *Neisseria meningitides* AP endonuclease, Nape and Ser178 in *E.coli* AP endonuclease, ExoIII) [31]. The equivalent residue in Nexo (an exonuclease of *N*.*meningitides*) is also a histidine (His167). Similar to our observations on XthA in this study, Nexo has also been demonstrated to be a very weak AP endonuclease [31]. Moreover, Carpenter *et al.* demonstrated that modification of His167 to Glycine or Serine resulted in a substantial increase in the AP endonuclease activity of Nexo, signifying the critical role of this histidine as a structural determinant for the AP endonuclease activity [31]. We hypothesize that the presence of histidine side chain at the abasic ribose binding site is involved in the impairment of the binding of abasic ribose to the enzyme which would prevent the consequent cleavage of the substrate, in turn leading to a low AP endonuclease activity of XthA. Additionally, in *M.leprae*, the pathogen causing leprosy, *xthA* has been converted to a pseudogene [6], further substantiating a less vital role of XthA in pathogenic mycobacteria.

In the ExoIII/Ape1/Apn2 family of proteins, Ec-ExoIII and human Ape1 have been extensively studied, and both of these enzymes have been shown to possess AP endonuclease and 3′→5′ exonuclease activities [38]. Our study demonstrates that XthA, the less active AP endonuclease of mycobacteria, displayed an efficient metal ion dependent 3′→5′ exonuclease activity on a large variety of substrates which includes: duplex DNA with 3′ recessed or blunt termini, duplex DNA with an internal nick or an AP site, as well as a single-stranded DNA, emphasizing the primary role of this protein as an exonuclease. On the other hand, *M.tuberculosis* End was observed to be competent to digest exonucleolytically, specifically a duplex DNA that comprised of a damaged AP site and not any other substrate employed in this study. Although the function of the 3′→5′ exonuclease activity of Exonuclease III family is unknown and has been considered redundant, the discovery of substantial exonuclease activity associated with Endonuclease IV homologs, in *E.coli* [36] and *M.tuberculosis* argues in favor of a significant functional importance for this activity *in vivo*.

The *in vitro* observations using purified enzymes and defined oligonucleotide substrates cannot be simply extrapolated to an *in vivo* situation without taking into account the complex cellular network. In *E.coli* crude cell extracts, Ec-ExoIII accounts for 90% of total AP endonuclease activity [10]. The measurement of AP endonuclease activity in the mycobacterial cell-free extracts of the parental and mutant strains of *end* or/and *xthA,* demonstrate End as the major apurinic endonuclease under normal growth conditions. However, the activity of XthA in these cultures was only conspicuous by its absence. To evaluate if the activity of XthA might be induced under DNA damaging conditions, logarithmically growing cultures of *M.tuberculosis* were subjected to treatment with DNA damaging agents such as methylmethane sulfonate (MMS), hydrogen peroxide (H_2_O_2_) or mitomycin C (MMC) at various concentrations (1– 5 mM MMS, 0.1– 1 mM MMC and 1 mM-10 mM H_2_O_2_) for 24 hours followed by determination of XthA activity in the cell free extract. Apart from *M.tuberculosis*, MtbΔ*end* was also employed in these studies so that even slight induction in the activity of XthA could be measured. However, inspite of exposure to these DNA damaging agents, we did not find any measurable activity of XthA even at the highest concentration of these DNA damaging agents used under our experimental conditions (data not shown). Based on the above results, End seems to represents the major AP endonuclease in *M.tuberculosis*. However, we cannot rule out the possibility that during the infection of the host by this pathogen there could be situations that could lead to induction of either End or XthA.

To investigate the role of these AP endonucleases in repairing the DNA damage and thereby protecting *M.tuberculosis*, we employed disc diffusion assay. The response of *M.tuberculosis* to DNA damage resulting from the exposure of reactive oxygen intermediates in mutant strains lacking one or both the AP endonucleases was measured by growth inhibition zone around a paper disc impregnated with CHP. At 5 mM and 10 mM concentrations of CHP, a significantly enhanced sensitivity was observed in the case of MtbΔ*end*Δ*xthA* strain in comparison to the parental strain. More importantly, the absence of End in *M.tuberculosis* (MtbΔ*end*) resulted in an enhanced sensitivity in comparison to the parental and MtbΔ*xthA* strains at 10 mM concentration of CHP. These results suggest that in the *M.tuberculosis* BER pathway, the removal of damaged DNA resulting from oxidative stress is primarily carried out by End. XthA that possesses only a weak AP endonuclease activity with a predominant 3′→5′ exonuclease activity plays a less significant role in the repair of such damage. More importantly, the absence of Endonuclease IV homolog in humans and the contribution of End as the major AP endonuclease in *M.tuberculosis*, opens up a new arena of research targeting this crucial enzyme for developing anti-tubercular molecules.

In summary, the present study provides the first evidence for the presence of active AP endonucleases in *M.tuberculosis* and for distinct substrate preferences of these AP enzymes. We demonstrate that End is not only a more efficient AP endonuclease enzyme than XthA but it also represents the major AP endonuclease activity in *M.tuberculosis* and plays a crucial role in defense against oxidative stress in comparison to XthA. In addition, while End possesses a metal ion independent exonuclease activity; XthA is a metal ion dependent enzyme which predominantly acts as a 3′→5′ exonuclease possessing weak AP endonuclease activity. The AP endonuclease activity of both the *M.tuberculosis* AP endonucleases is stimulated by Mg^2+^ and Ca^2+^ and displays a preferential recognition for abasic site paired opposite to a cytosine residue in DNA. In our future studies, we aim to elucidate the biological implications of AP endonuclease activity in the pathogenesis of *M.tuberculosis* by employing single and double mutant strains of *end* and *xthA* in animal models.

## Supporting Information

Table S1 Primers used for cloning *end* and *xthA* genes in pet-21c(+) vector for purification of the encoded proteins. (PDF)
