# Supplementary material for: Endonuclease IV Is the Major Apurinic/Apyrimidinic Endonuclease in Mycobacterium tuberculosis and Is Important for Protection against Oxidative Damage
Source: PLoS One. 2013 Aug 1;8(8):e71535. doi: 10.1371/journal.pone.0071535 (PMC3731287; doi:10.1371/journal.pone.0071535)
Supplement: Table S1 — (PDF) [file pone.0071535.s001.pdf]

Table S1. Primers used for cloning *end* and *xthA* genes in pet-21c(+) vector for purification of the encoded proteins.

Primer	Sequence (5´→3´)
F-End-*Nhe*I-EcoRI	ggatccgctagcgaattcgtgctcattggttcgcatg
R-End-Strep-*Hind*III	gaattcaagcttattatttttcgaactgcgggtggctccaagcgctgctgccttctttcccgcag
F-XthA-*Nhe*I-*BamH*I	gaattcgctagcggatcccccgacggcacaattgac
R-XthA-Strep-*Hind*III	gaattcaagcttattatttttcgaactgcgggtggctccaagcgctcccggcgtgcaggtcgacgag

Relevant restriction sites are underlined.
